# Barriers and facilitators to parents seeking and accessing professional support for anxiety disorders in children: qualitative interview study

**DOI:** 10.1007/s00787-018-1107-2

**Published:** 2018-01-25

**Authors:** Tessa Reardon, Kate Harvey, Bridget Young, Doireann O’Brien, Cathy Creswell

**Affiliations:** 1 School of Psychology and Clinical Language Sciences, University of Reading, Reading, UK; 2 Department of Psychological Sciences, University of Liverpool, Liverpool, UK

**Keywords:** Help seeking, Barriers, Parents, Children, Anxiety disorders

## Abstract

Anxiety disorders are among the most common mental health disorders experienced by children, but only a minority of these children access professional help. Understanding the difficulties parents face seeking support for child anxiety disorders could inform targeted interventions to improve treatment access. The aims of the study were to identify barriers and facilitators to seeking and accessing professional support for child anxiety disorders, and ways to minimise these barriers. A qualitative interview study was conducted with parents of 16 children (aged 7–11 years) with anxiety disorders identified through screening in schools. Barriers and facilitators were identified in relation to four distinct stages in the help-seeking process: parents recognising the anxiety difficulty, parents recognising the need for professional support, parents contacting professionals, and families receiving professional support. Barriers and facilitators at each stage related to the child’s difficulties, the role of the parent, and parent perceptions of professionals and services. Findings illustrate the need (1) for readily available tools to help parents and professionals identify clinically significant anxiety in children, (2) to ensure that families and professionals can easily access guidance on the help-seeking process and available support, and (3) to ensure existing services offer sufficient provision for less severe difficulties that incorporates direct support for parents.

**Electronic supplementary material:**

The online version of this article (10.1007/s00787-018-1107-2) contains supplementary material, which is available to authorized users.

## Introduction

Anxiety disorders are among the most common mental health disorders experienced across the lifespan [1] and are associated with significant negative outcomes for individuals [2, 3] and economic burden for society [4]. These typically first emerge during childhood, with a median age onset of 11 years [1] and affect approximately 6.5% of children and adolescents [5]. Effective treatments for child anxiety disorders exist (e.g. cognitive behaviour therapy; CBT) [6] with clear evidence for the lasting benefits both in alleviating anxiety [7, 8] and reduced risk of other mental health difficulties [9, 10]. However, rates of access to treatment for childhood mental health difficulties are poor [11, 12] and approximately two-thirds of children with anxiety disorders do not access any professional help [13].

Poor rates of treatment access for childhood mental health problems in the UK have been linked to limited service provision [14]. The extent of service provision, however, is only part of the story and strategies to improve provision must take the broader picture into account. Children with mental health problems typically depend on a parent or caregiver to seek help on their behalf. Studies focusing on parents’ perceptions of seeking professional help for child mental health difficulties highlight a broad range of difficulties families can face seeking professional support, including structural issues associated with mental health services, as well as attitudinal barriers and a lack of knowledge surrounding mental health and the help-seeking process [15]. However, previous studies examining parental help seeking have tended to focus on populations of service users, and therefore the difficulties experienced by those who have not reached services are less well understood. Moreover, little is known about help seeking for difficulties relating to child anxiety disorders specifically. Given the early onset, high prevalence and low rates of treatment access for anxiety difficulties (compared with, for example, behavioural difficulties [12]), it is important to establish the barriers to treatment faced by these families in particular. Indeed, among adults, anxiety disorders are associated with lower rates of recognition [16] and longer delays in receiving treatment [17] compared to other mental health disorders. This evidence that there are specific barriers to help seeking for anxiety difficulties later in life raises questions about whether these barriers may also apply to children and young people. An improved understanding of the factors that help or make it harder for families to seek professional help specifically in the context of an anxiety disorder would inform targeted interventions to improve rates of treatment access.

This study aimed to (1) identify barriers and facilitators to seeking and accessing support from professionals for anxiety disorders among parents of children with an anxiety disorder identified in the community; and (2) identify ways to overcome and minimise barriers to seeking and accessing professional support. Given the limited understanding of help seeking for child anxiety problems, an inductive qualitative approach was used to provide a detailed insight into the barriers/facilitators described by these families.

## Method

The study was approved by the University of Reading Research Ethics Committee (UREC 15/04), and participants provided informed consent.

### Recruitment

The study aimed to recruit a community sample of parents of children with an anxiety disorder with a diverse range of experiences. As there is regional variation in available support for child mental health difficulties, and mental health support provided within schools is also likely to vary, we aimed to include families from a range of geographic locations and schools within England. A two-stage screening process was used. Firstly, during the period March to October 2015, 102 primary/junior schools from different geographic locations in England and with a varied demographic profile were invited to participate. Ten schools were recruited across seven geographic locations (Buckinghamshire, East Sussex, Hampshire, Middlesex, Northumberland, Surrey, Worcestershire) and included nine state schools (2.1–57.5% of children on the roll receiving free school meals) and one fee-paying school.

**Fig. 1:**
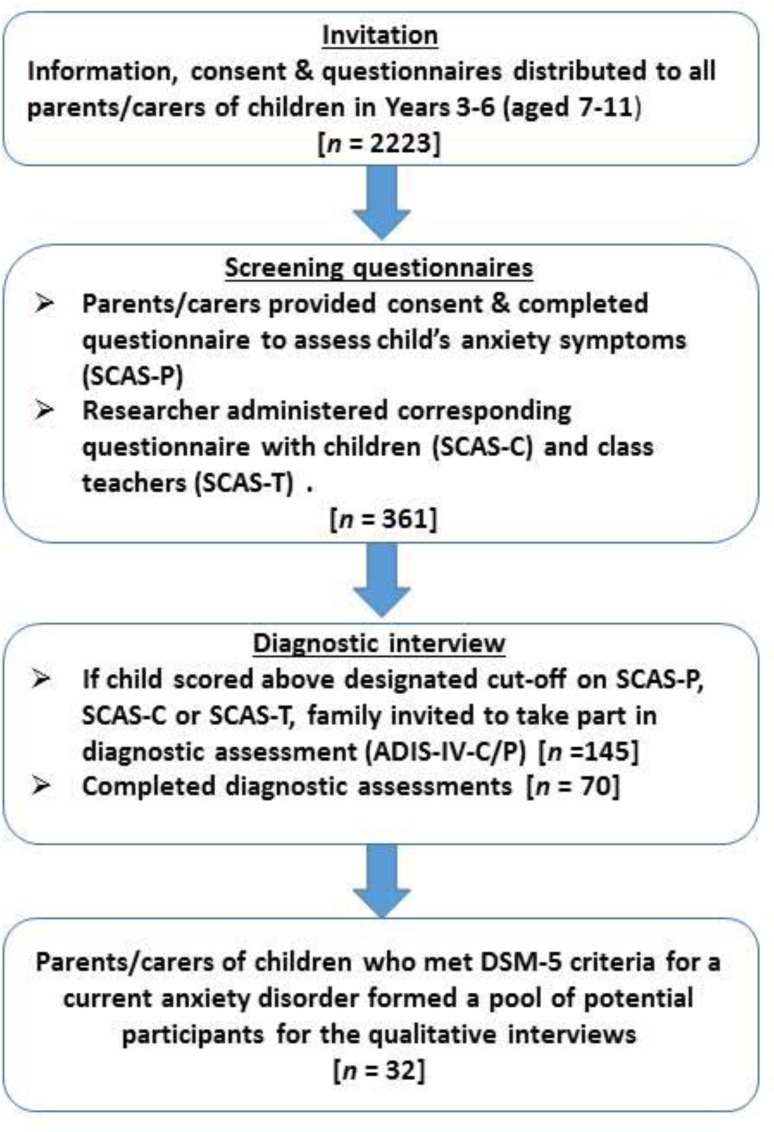
Screening process within recruited schools to identify potential participants

The screening process within recruited schools is detailed in Fig. 1. Within recruited schools, study information and consent/questionnaires were distributed to all parents with a child in years 3–6 (aged 7–11 years). Parents were asked to complete a questionnaire to assess their child’s anxiety symptoms (SCAS-P), and one or two researchers attended the school to administer the corresponding questionnaires (SCAS-C and SCAS-T) with the children and class teachers of those children whose parent provided consent. In cases where the child scored above the designated cutoff^1^ on either the SCAS-P, the SCAS-C, or the SCAS-T, the family was invited to take part in a follow-up diagnostic assessment (ADIS-IV-C/P). Following the diagnostic assessment, parents were sent a report summarising the assessment findings, which where applicable described the particular difficulties with anxiety that the child was experiencing. Parents of the children who met the DSM-5 criteria for a current anxiety disorder formed a pool of potential participants for the qualitative interviews. Schools and families were reimbursed for giving their time to participate in the study. Schools were given £5 for each set of complete parent/child/teacher questionnaires, and families were given a £20 gift voucher for taking part in the diagnostic assessment, and a further £20 gift voucher for taking part in the qualitative interview.

### Measures

#### Spence Children’s Anxiety Scale-Child (SCAS-C/P)

The SCAS-C/P are child and parent report questionnaires and comprise 38 items addressing symptoms of DSM anxiety disorders (and 6 filler items in the child report version). Items are rated on a four-point scale (never = 0; sometimes = 1; often = 2; always = 3), and total scores reflect the sum of responses to the 38 anxiety items. The SCAS-C/P are widely used measures of child anxiety symptoms, with good evidence in support of their reliability and validity [18–21], and excellent internal consistency in the current sample (SCAS-C *α* = 0.95; SCAS-P *α* = 0.91).

#### Adapted Spence Children’s Anxiety Scale-Teacher (SCAS-T)

The research team developed a teacher-report version of the SCAS-C/P. Eight items that appear on the SCAS-C/P were considered inappropriate for teachers (e.g. ‘I would feel afraid of being at home on my own’) and omitted. The wording of the remaining items was amended to reflect the reporter (e.g. ‘I worry about things’ was changed to ‘Worries about things’). The SCAS-T comprised 30 items, with the same four-point response scale as the SCAS-C/P, and total scores reflect the sum of responses to all items. Internal consistency for the SCAS-T in the current sample was excellent (*α* = 0.90).

#### Anxiety Disorders Interview Schedule-IV-Parent and Child Interview (ADIS-C/P)

The ADIS-C/P consists of structured parent and child interview schedules consistent with DSM-IV anxiety diagnoses and common comorbid diagnoses, and has strong psychometric properties [22]. Minor amendments were made to the interview schedules to enable diagnoses consistent with DSM-5 diagnostic criteria. The child and parent interviews were administered independently, with diagnoses and clinical severity ratings (CSRs) assigned independently for each interview. As per the guidelines, a child met diagnostic criteria where the required symptoms were reported and a CSR from 4 to 8 was assigned. In cases where there were discrepancies between the child and parent report, the higher CSR was assigned as the overall CSR. With the exception of two interviews, all assessments were administered by one assessor (TR), and for the first 20 assessments, interviews were discussed with an experienced clinician (CC) and a consensus reached. Assessor reliability was checked at this stage (with a minimum kappa/ICC of 0.85 required), and subsequently one in six interviews were discussed and consensus reached. Overall inter-rater reliability within the assessment team was excellent (child report diagnoses, kappa = 0.95; CSR ICC = 0.97; parent report diagnoses, kappa = 0.98, CSR ICC = 0.96).

### Participants

We used purposive sampling to ensure that those families invited to be interviewed (from the pool of 32 eligible families) varied in their experiences surrounding seeking help for their child. Invited families varied on the following characteristics, judged by the researchers to be relevant to their help-seeking experiences: (1) child age and gender; (2) type and severity of child anxiety disorders, (3) prior help seeking for the child’s anxiety difficulties, (4) socioeconomic status, and (5) geographic location.

Parents of 20 children were identified. Interviews were conducted with 16 of them; at which point analyses indicated that theoretical saturation [23] had been reached, as new data ceased contributing to the development and refinement of new codes and concepts. Participant characteristics are provided in Table 1. Interviews were conducted with parents of 11 girls and 5 boys, aged 7–11 years (median age 8.9 years). Interviewees were typically mothers (14 families), but in two cases the father was also interviewed. The sample spanned socioeconomic status; with nine families categorised as higher/professional and three received free school meals for their children. The sample was predominantly white British (*n* = 13), and three children were from other white backgrounds. Children had between one and three anxiety disorder diagnoses, including separation anxiety disorder (*n* = 3), social anxiety disorder (*n* = 5), generalised anxiety disorder (*n* = 10) and specific phobias (*n* = 7), with clinical severity ratings (CSRs) for primary disorders ranging from 4 to 6. Children had a non-anxiety comorbid disorder in one-quarter of cases (depression, *n* = 1; attention deficit hyperactivity disorder, *n* = 2; oppositional defiant disorder, *n* = 1). There was variation in prior help seeking reported across cases (sought help/advice from a professional, *n* = 9; not sought help/advice from a professional, *n* = 7).

**Table 1:** Characteristics of the participants

Child	
*n*	16
Median age (range), years	8.9 (7.7–11.7)
Female, *n* (%)	11 (68.8)
Parent^a^	
*n*	18
Median age (range), years	43.5 (25–54)
Mother, *n* (%)	16 (88.9)
SES	
Free school meals	
*n* (% of families)	3 (18.8)
Higher/professional^b^	
*n* (% of families)	9 (56.3)
Child’s ethnicity	
White British	13 (81.3)
Other white background	3 (18.8)
ADIS primary anxiety diagnosis *n* (%)	
Separation anxiety disorder	2 (12.5)
Social anxiety disorder	1 (6.3)
Generalised anxiety disorder	9 (56.3)
Specific phobia	4 (25.0)
Primary anxiety diagnosis CSR *n* (%)	
CSR 4	10 (62.5)
CSR 5	4 (25.0)
CSR 6	2 (12.5)
Presence of anxiety and other disorders (based on ADIS) *n* (%)	
Separation anxiety disorder	3 (18.8)
Social anxiety disorder	5 (31.3)
Generalised anxiety disorder	10 (62.5)
Specific phobia	7 (43.4)
Major depressive disorder	1 (6.3)
ADHD	2 (12.5)
ODD	1 (6.3)
Parent reported contact with GP and/or school staff for help or advice related to child’s anxiety difficulties	9 (56.3)
Parent reported child had received referral to CAMHS (for anxiety or other difficulties)	6 (37.5)

*SES* socioeconomic status, *ADIS* Anxiety Disorders Interview Schedule, *CSR* clinical severity rating, *CAMHS* Child and Adolescent Mental Health Service

^a^Two interviews were conducted with the child’s two parents

^b^Higher/professional = managers, directors, senior officials, professional occupations, based on the Office for National Statistics Standard Occupation Classification

### Procedure

Semi-structured topic-guided interviews were conducted with parents, with 4 interviews conducted face to face and 12 on the telephone. Interviews were conducted by a doctoral researcher (TR) and lasted from 33 to 79 min. Interviews were audio-recorded and explored parents’ views and experiences of recognition and help seeking for their children’s anxiety difficulties. The topic guide explored parents’ (1) knowledge and beliefs surrounding child anxiety; (2) recognition of their child’s anxiety difficulties; (3) knowledge and beliefs surrounding help seeking; (4) beliefs and experiences of services; and (5) suggestions for improvements to the help-seeking process; but it was used flexibly, allowing for variation in the order and wording of questions and ensuring participants had the opportunity to discuss issues that departed from the prepared areas of questioning. Interviews were transcribed verbatim, with all information that could identify participants removed, and participants’ names replaced with pseudonyms.

### Coding and analysis

Analysis of the transcribed interviews was guided by the six phases of a thematic analysis described by Braun and Clark [24]. The analysis was inductive, in that codes and themes were data driven, rather than according to a pre-existing theoretical framework and focussed on the specific research questions of the study, rather than attempting to encompass the entire content of the dataset. Codes were generated through an iterative process, whereby as each new transcript was coded, earlier transcripts were revisited and codes were constantly reviewed and refined. Codes were gradually organised into candidate themes and sub-themes, paying particular attention to linkages and distinctions between key ideas and concepts, and commonalities and discrepancies within and across transcripts. The analysis was led by one researcher (TR) who met regularly with other research team members to discuss codes and candidate themes, and alternative interpretations of the data. Following team discussions, candidate themes and sub-themes were reviewed and refined to ensure the thematic structure provided a coherent and credible interpretation of the data that did not only reflect a single researcher’s perspective.

## Results

**Fig. 2:**
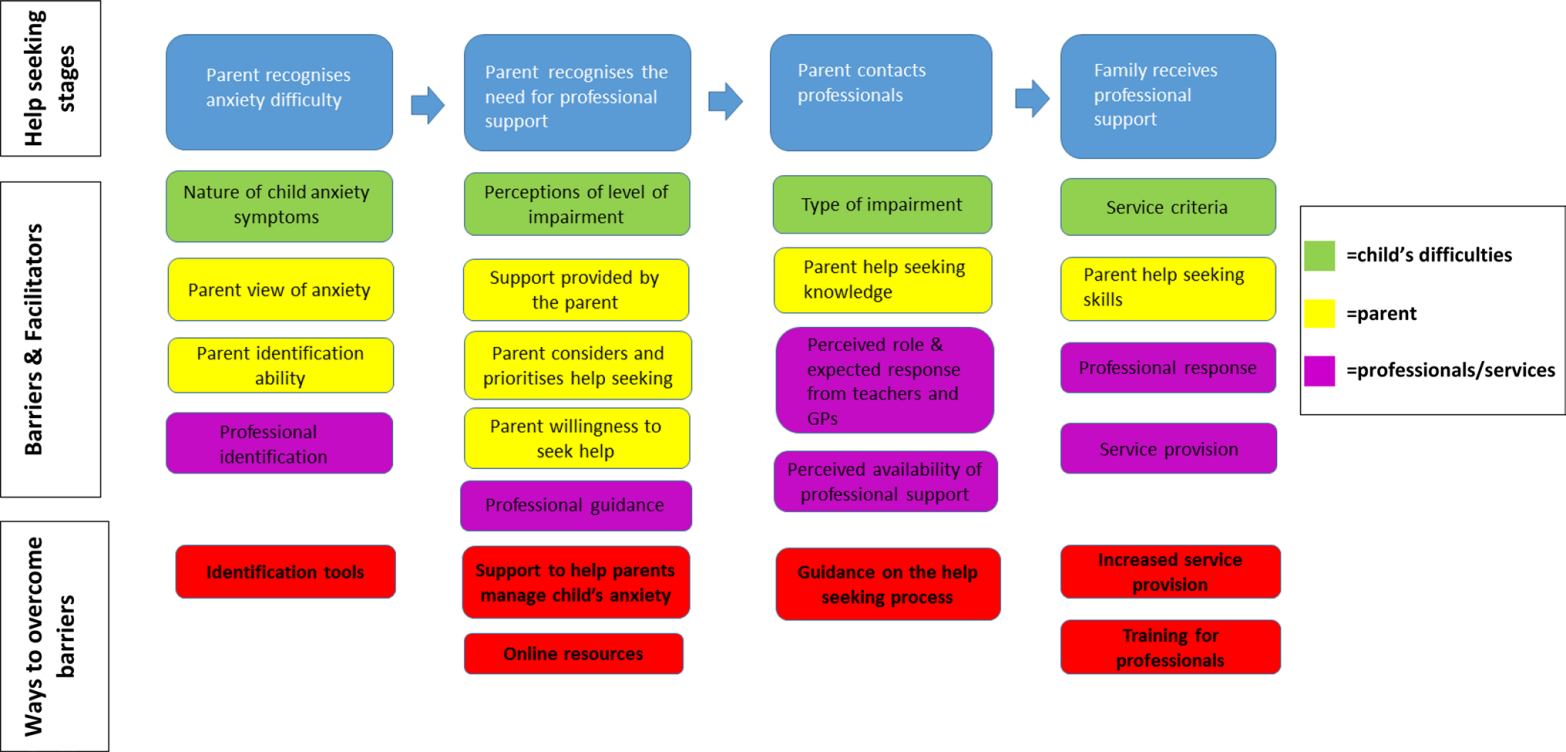
Barriers and facilitators at four key stages of the help-seeking process

The findings are described in relation to the four main themes identified which correspond to key stages in the help-seeking process: (1) parent recognises anxiety difficulty; (2) parent recognises the need for professional support; (3) parent contacts professionals for help or advice; and (4) family receives professional support to help manage and overcome a child’s difficulties with anxiety. As illustrated in Fig. 2, a number of factors were identified that helped or hindered families at each stage and influenced whether a family successfully progresses towards receiving treatment. Barriers and facilitators identified at each stage in the help-seeking process related to (1) the child’s difficulties, (2) the parent, and (3) parent perceptions of professionals and services; and parents also suggested ways to overcome barriers associated with each stage. Findings are described in detail (including direct quotations from the interviews) in Supplementary Material 1, and a summary is provided below.

### Parent recognises anxiety difficulty

Parents faced difficulties in both identifying their child symptoms or behaviour as ‘anxiety’, and determining whether or not these symptoms or behaviour were a ‘difficulty’. Parents more readily attributed some symptoms (e.g. clingy or nervous behaviour) to anxiety than other symptoms (e.g. temper tantrums or anger outbursts); and found it easier to identify sudden or marked changes in a child’s behaviour as problematic, compared to more gradual or fluctuating changes in behaviour. A perception that anxiety is a personality trait or a common childhood experience also deterred parents from considering their child’s anxiety as a problem.

Parents were clear that it was their responsibility to make judgements about the extent or severity of a child’s anxiety, but some lacked confidence in their ability to do this. They reported drawing on their own and others’ experience when forming judgements about whether their child’s anxiety was ‘normal’ or not; and this helped some identify their child’s anxiety difficulties, but deterred recognition for others. Interestingly, while parental anxiety helped some parents recognise similar difficulties in their child, others parents were concerned about being an oversensitive or overprotective parent and this hindered recognition. As well as parents having a key role in identifying a child’s anxiety difficulties, the parents also described the important role that professionals can also play either in validating (or failing to validate) parental concerns, or in raising (or failing to raise) concerns with parents.

### Parent recognises the need for professional support

Perceptions surrounding the negative impact associated with a child’s anxiety prompted (or deterred) parents to recognise the need for professional support. The importance parents attached to their own role as the primary source of support for their child was evident, and parents varied in confidence in their ability to manage their child’s anxiety. Knowing that the support they could provide their child may be insufficient prompted some parents to recognise that additional input from a professional may be needed.

Some parents contemplated the possibility of help seeking for some time before actually seeking help. The general busyness of life lengthened or hindered this process of contemplation, while for others, changes in circumstances or the nature of a child’s difficulties elevated help seeking from a possibility to a priority. Equally, similar to the role for professionals in helping parents identify a child’s anxiety difficulties, some parents also identified a role for professionals in helping parents determine whether further support from professionals to address a child’s anxiety was required or not.

Parents’ views surrounding (1) the potential benefit and appropriateness of anxiety treatment; (2) their child’s willingness or reluctance to seek help; and (3) sharing concerns with others also each influenced their willingness to seek professional help for their child. Importantly, parent perceptions surrounding the stigma associated with anxiety and mental health were important determinants of their openness (or reluctance) to share concerns about their child, both informally and with professionals. Parents were concerned that other people would blame their parenting for their child’s difficulties, and they were also concerned about the negative consequences for their child, if they talked to other people about their child’s difficulties.

### Parent contacts professionals

Parents reported lacking knowledge of how to seek help for their child and were uncertain about who to contact for help and advice. Similar to earlier recognition stages, parents drew on their own and others’ experiences to determine where and how to seek help. Parents varied in their views of whether GPs or teachers were the most appropriate point of initial contact, illustrating a key role for both. Parents also described anticipating how GPs and teachers would respond, and parents’ perceptions of the anticipated response, and the family’s relationship with these professionals, influenced their decision to contact (or not contact) a professional for help or advice. In particular, anticipating that professionals may dismiss their concerns or blame them as a parent deterred some parents from seeking help. Interestingly, some parents described a lack of available services and long waiting times for child mental health difficulties and commented that this had deterred them from making initial contact with a professional.

### Family receives professional support

Parents who had sought help from professionals described difficulties meeting strict service criteria and limited or lacking service provision as barriers to accessing support to help overcome their child’s anxiety difficulties. Among families who had sought help, the key role that both parents and professionals could play in determining whether a family receives support or not was also apparent. In particular, parents described the importance of knowing how to communicate with professionals, and the need to make repeated contact with professionals and not give up; as well as the importance of the response from individual professionals.

## Discussion

Barriers and facilitators associated with four distinct stages in the help-seeking process were identified from interviews with parents of 16 children with anxiety disorders. This study illustrates how challenging it can be for parents to identify and make judgements about the extent or severity or a child’s anxiety. Notably, several recognition barriers were anxiety specific, including the perception that anxiety is a personality trait or common childhood experience and the role of parents’ own experience (or lack of experience) of anxiety. Indeed, complexities surrounding the role of parental anxiety indicated here help account for discrepant findings across studies examining child mental health service use, with evidence indicating both the potential positive [25] and negative [26] impact of parental anxiety on child service use.

Recognition of the need for help is a key determinant of help seeking for mental health difficulties in adults [27], and findings reported here illustrate the range of factors that influence whether or not a parent recognises and prioritises the need for professional help for their child’s anxiety difficulties. We found that parents’ views surrounding treatment and the stigma associated with anxiety difficulties can contribute to parental reluctance to seek help. This echoes findings across the broader literature surrounding mental health difficulties in children [15], and illustrations of the impact of stigma on parenting [30] and help seeking among adults with mental health difficulties [31]. Similarly, in relation to the subsequent stage of contact with professionals, the barrier posed by a lack of available information surrounding the help-seeking process and the importance of the anticipated response from professionals are also reported elsewhere [15]. Importantly, structural barriers associated with services, including a lack of available services and high demands on services, appeared most influential following initial contact with professionals, and influenced whether a family accessed professional support or not.

### Implications

This study identifies areas for intervention and ways to overcome key barriers to seeking and accessing professional support for child anxiety disorders (see Fig. 2). In relation to improving recognition of child anxiety disorders, findings highlight the importance of raising awareness about the presentation of anxiety difficulties in children. In particular, parents’ experiences identify a need for readily available tools to help families, teachers, and GPs to make judgements about when a child experiencing anxiety may benefit from professional support, and to differentiate between developmentally appropriate fears and worries, and clinically significant levels of anxiety. Indeed, GPs have similarly identified a lack of available tools to help identify anxiety difficulties in children [28]; although questionnaire measures designed to identify anxiety symptoms in children exist (e.g. the SCAS), these tools are long and time consuming to complete, often making them impractical for use in school or primary care settings [29]. Furthermore, as reported elsewhere [15], the need to raise awareness of and ensure families can access guidance on the help-seeking process is also apparent. The findings indicate the particular need for efforts to reduce the stigma associated with child mental health difficulties and negative attitudes towards parents whose children experience difficulties. It is important that guidance is readily available to families and professionals working with families (e.g. GPs and teachers) to ensure that they are aware of and understand: (1) the professional points of contact and support available; (2) the benefits of professional support and early intervention for anxiety difficulties; (3) tools and strategies available to help support a child experiencing difficulties with anxiety. Efforts to promote public awareness of appropriately endorsed online resources could help to ensure improved access to such information and guidance. Indeed, evidence of the need for greater awareness of and access to guidance for professionals is consistent with the findings that GPs themselves feel ill equipped to manage child anxiety disorders [28].

Additionally, findings surrounding parents’ experiences also highlight the importance of ensuring available services for child anxiety disorders include (1) sufficient provision that incorporates early intervention for less severe difficulties; (2) direct support for parents to enable them to help their child manage and overcome their difficulties with anxiety; and (3) training and guidance for professionals (e.g. GPs and teachers) to equip them with the skills to communicate effectively with families. Indeed, these findings have clear implications for access to child and adolescent mental health services more broadly, and indicate important areas to target to improve access to these wider services.

### Strengths and limitations

By identifying children in the community with anxiety disorders, this study explored the views and experiences of families who varied in their prior help seeking, including those who had not sought or accessed professional help. This is a notable departure from much of the existing work examining help seeking for child mental health difficulties in which families tend to be recruited through specialist support services and comprise solely those who have successfully accessed professional help. The purposive sampling approach ensured that the sample had a varied socioeconomic profile and included families from different schools and geographic regions in England. Moreover, parents’ experiences surrounding seeking professional support is likely to change as a child gets older, and by focusing specifically on anxiety disorders among pre-adolescent children our findings provide insight into help seeking among a population for whom parents play a particularly pertinent role. Furthermore, focusing on pre-adolescent children means that findings can be used to inform targeted interventions to improve access to professional support, specifically among this population for whom rates of access are particularly poor.

Nevertheless, it is important to note that there was a likely participation bias in the study. Many families invited to take part in the initial screening stage of the study did not participate, and approximately 50% of those invited to take part in follow-up diagnostic assessments did not complete the assessment. Although parents who had concerns about a child’s anxiety were not specifically targeted, it is likely that these families were overrepresented in our sample. Other barriers (e.g. the parent did not speak English) are likely to have prevented some families from taking part, and the sample was predominantly white British. This means that the views and experiences among underserved families, including families from minority ethnic backgrounds, and parents who had not considered the possibility that their child may be experiencing anxiety, are unlikely to have been fully captured in this study. Similarly, many schools who were invited to take part in the study did not respond, and it is possible that schools with staff who had some awareness or understanding of anxiety difficulties in children were more likely to take part, and therefore the experiences of families from schools where staff have no awareness of child anxiety may not have been captured. Furthermore, the study’s capacity to identify children in the community with anxiety disorders was limited by the tools currently available. Although the SCAS is a widely used measure of anxiety symptoms in children, and three informant versions were used in this study, its capacity to identify children with clinically significant levels of anxiety has not been established. Therefore, the number of parents of children with anxiety disorders who were missed (i.e. the child scored below the cutoff score on the SCAS-P, SCAS-C and SCAS-T) is not known.

It is also important to acknowledge that other aspects of the methodology will also have shaped the data in this study. One researcher (TR) conducted the interviews and led the analysis, and this researcher also administered screening questionnaires and diagnostic assessments with families in the recruitment phase. TR’s knowledge of participating families prior to the qualitative interviews and participants’ awareness of TR’s position as the study lead with expertise in anxiety assessments will also have influenced the interview data. During team meetings to discuss codes and emerging themes, the role of TR’s prior contact with participants was reflected upon and alternative potential interpretations of the data were carefully considered. The fact that prior to the qualitative interview, families took part in an anxiety assessment for their child and received a report summarising the difficulties their child was experiencing with anxieties will also have shaped the qualitative interview data. Indeed, as described earlier, for some parents, taking part in the study influenced their views surrounding seeking help for their child and some of the study findings.

This study importantly underlines the range of challenges families face throughout the help-seeking process from child anxiety disorders and identifies key interventions needed to minimise these challenges and ensure more families seek help and go on to access professional support. Closer examination of the particular barriers to seeking help for childhood anxiety disorders among underserved populations is also necessary.

## Electronic supplementary material

Below is the link to the electronic supplementary material.

Supplementary material 1 (DOCX 43 kb)

## Footnotes

1 Published cut scores on the SCAS-C indicating ‘elevated’ anxiety symptoms (for girls and boys aged 8–11 years) were used (available on http://www.scaswebsite.com). Published cut scores are not available for the SCAS-P or SCAS-T; therefore, the following were used as cut scores: SCAS-P mean total score + 1 s.d in a normal sample as published on http://www.scaswebsite.com; SCAS-T mean total score + 1 s.d in the current study.
