# Supplementary material for: Barriers and facilitators to parents seeking and accessing professional support for anxiety disorders in children: qualitative interview study
Source: Eur Child Adolesc Psychiatry. 2018 Jan 25;27(8):1023–31. doi: 10.1007/s00787-018-1107-2 (PMC6060962; doi:10.1007/s00787-018-1107-2)
Supplement: Supplementary file 1 — Supplementary material 1 (DOCX 43 kb) [file 787_2018_1107_MOESM1_ESM.docx]

Supplementary Material 1

Results

1. Parent recognises anxiety difficulty

1.1 Nature of child anxiety symptoms

Both the development or course of a child’s anxiety difficulties, and the type of symptoms a child displayed influenced whether parents i) attributed a child’s behaviour or symptoms to ‘anxiety’ or not; and ii) perceived the anxiety to be a problem or not. Some parents described that their child had displayed anxieties from a very young age, and clearly labelled their child as anxious (‘*she’s always when she’s gone into a new environment when she was younger she was very clingy she wouldn’t let go she was crying she took a long time to settle in*’ [1036]). Others described the anxiety developing over a period of time, and this gradual emergence of particular behaviours or symptoms hindered or delayed recognition for some parents (‘*it happened gradually it didn’t happen you know from one day to another suddenly it gradually I think creeped in you know’* [ID1091]). In contrast, some parents referred to more marked changes in their child’s behaviour or specific events that either triggered their child’s anxiety or marked a crisis point, and acted as recognition prompts:

*‘compare her to year 3 from when she was in year 4 was there was just two completely different children really’* [ID 1212]

‘*for us it was I suppose it was a bit easy because it went from nothing to everything because of her accident’* [ID 7]

For some parents the persistence of these ‘new’ behaviours led them to consider the behaviour as problematic (‘*he has refused to* [leave home] *pretty much constantly since about April this year’* [ID 1131). Whereas other parents described that their child’s anxiety fluctuated or anticipated that it would not continue, and therefore did not consider their anxiety as problematic:

*‘he’s not doing that now erm I think it was sort of a period that he was going through I think and he seems to have settled down’* [ID 1020]

*‘I think that Michelle’s going to come out of it’* [ID 3]

Parents who felt that their child had always been anxious often described them as shy or nervous or clingy in new situations; whereas parents who considered their child to be socially confident, felt the anxiety was less obvious and hard to identify (‘*she er in some ways is a very chatty kind of you know quite a bubbly personality erm and not the kind of shy retreating child in in the back of the classroom’* [ID 53]). Parents also experienced difficulties distinguishing between anxiety and other comorbid difficulties (*‘it’s so tangled up in his other aggressive behaviours …from very young there were behaviours but I couldn’t have said they were anxiety related*’ [ID 1209]); and expressed uncertainty surrounding whether their child’s behaviour problems or anger outbursts were indicators of anxiety or not:

‘*sometimes I think it does come down to just a temper tantrum rather than it being a real worry’* [ID 2011]).

*‘a withdrawn type of quiet insular sort of reaction which is what I would have expected from anxiety rather than the complete explosive* [behaviour]*’* [ID 7]

Several parents referred to physical symptoms as key signs of a child’s difficulties (‘*I think it was just stress and emotion he had eczema all over his foot which he’d never done before*’ [ID 1205]). Other parents similarly identified the absence of physical symptoms or a child’s limited capacity to understand and articulate their difficulties as obstacles to recognition (‘*if you ask Chloe why why is she feeling worried she doesn’t understand why she’s feeling worried*’ [ID 1212]). Changes in how a child expressed their anxiety as they got older helped some parents identify their child’s difficulties (‘*when she’s distressed now she vocalises it’* [ID 2]), and made it harder for others (‘*the anxiety is there but I think he er doesn’t outwardly manifest it as he did*’ [ID.1209]).

1.2 Parent view of anxiety

Several pertinent views surrounding the nature of anxiety and what it represents appeared to influence whether parents considered their child’s anxiety as a difficulty or not. It was apparent that some parents viewed anxiety as a personality trait or a fixed characteristic, and had not considered that anxiety was a problem that could be or needed to be addressed:

‘*you’ve always got in groups the loudest one the very you know extroverted one and the one that’s a bit more shy or a bit more in the background…there’s different types of people*’ [ID.3]

*‘I hadn’t seen it as such a diagnosable erm issue maybe I thought it was you know either your child has these characteristics or they don’t’* [ID. 2009]

Parents who did not consider their child’s anxiety as problematic, also described anxiety as a common experience among children or as part of growing up:

*‘as children develop they change…you know some things they do sort grow out of’* [ID 1020]

*‘I suspect that it’s it’s quite common for the younger ones to er to er have this…. I see it as part of growing up er you know the shyness’* [ID 3]

On the other hand, some parents who had considered the possibility that their child’s anxiety may be a difficulty, also acknowledged that anxiety could be a type of emotional or mental health difficulty (‘*I think people have all sorts of different* [mental health] *problems you know and anxiety is one thing they might be you know…anxiety is really one of of many problems’* [ID 1091]).

1.3 Parent identification ability

A perception that it is a parent’s role and responsibility to identify anxiety difficulties in children was evident across interviews. A number of parents’ lacked confidence in their ability to make judgments about whether their child’s anxiety was ‘normal’ or not (‘*they’re normal child childlike you know personality traits all children get nervous and anxious about things but is it too much or is it just you know regular*’ [ID.1131]). Some parents who reported experiencing anxiety themselves felt this experience helped them to identify similar difficulties in their child (‘*that’s why I can also see erm Ella’s anxieties I can kind of er have empathy for her because I I was there’* [ID1036]); but others were concerned that their own anxieties made them over sensitive or likely to over react to signs of anxiety in their child (*‘maybe I think about it a bit sooner than perhaps other people might erm sometimes cos I think he might be a bit like me and also his dad’s quite a nervous person so I think oh perhaps he’s going to be like me or his dad’* [ID 1020]). Equally, one parent felt that their lack of personal experience with anxiety made it harder for them to notice their child’s anxiety (‘*because it didn’t match either my experience or my husband’s experience…we didn’t have that comparison*’ [ID 1091]).

Parents also described making comparisons with other children when forming judgements about their child’s anxiety and, to varying degrees, referred to seeking advice from family, friends, other parents, colleagues and the internet to help them to make judgements about their child’s anxiety. While this process of informal help seeking helped some parents identify their child’s difficulties, for others it acted as a deterrent to parental recognition (*‘it just seems there’s probably quite a few….it seems that each child is experiencing something of a similar nature if you like’* [ID 38]). Several parents reported that this study provided an opportunity to help them establish the extent or severity of their child’s anxiety (‘*it was certainly a couple of the questions you asked* [in the questionnaire] *that helped me to realise that actually you know there might be a problem here*’ [ID 1036]; and parents who lacked confidence in their identification ability referred to the need for tools to help parents with this process (*‘like a checklist for er parents to look at is your child doing this or is your child acting like this maybe that’s the sign to worry’* [ID 1131]).

1.4 Professional identification

It was evident that some parents saw a role for professionals, as well as parents, in identifying anxiety difficulties in children, and one parent described the failure of a professional to raise concerns about their child as the reason for not considering the anxiety as a significant problem. Equally, a professional raising concerns with a parent, or a professional endorsing parental concerns, were key prompts to recognition for some parents (‘*the turning point was when the school actually came to me and said we’ve noticed behaviour changes’* [ID.1051]).

2. Parent recognises the need for professional support

2.1 Perceptions of level of impairment

Recognition that a child’s anxiety had a significant negative impact on the child’s life (‘*he got to the point where he didn’t want to go to school*’ [ID.1205]), or was distressing for the child (‘*it starts to affect them or it’s really it really erm it starts to be a burden and it builds up over time but then when it happens every night and it’s really something that he’s not happy with’* [ID 1091]) or caused significant interference in family life (‘*it’s soul destroying as a parent having a child like that I just it was making our lives miserable*’ [ID.53]) were described as key indicators of the need for professional support. Equally, a judgement that a child’s anxiety was not currently impacting on a child’s life was cited as a reason for not seeking professional help (‘*I don’t think it’s a serious issue I see it as erm er a small challenge she’s she’s shy I don’t think it causes her disruption to any degree’* [ID 3]). Judgements surrounding the potential impact for the child in the future also influenced parents’ perceptions of the current need for professional involvement (‘*I really don’t want to go into that situation* [puberty] *with the anxiety that we’ve got at the moment*’ [ID 2]).

2.2 Support provided by the parent

Parents commonly considered it their role and responsibility to help their child to manage their anxiety, and this appeared to deter some parents from recognising the potential for professional involvement. Parents described providing support for their child, and referred to seeking advice from family, friends, colleagues and through online forums to identify strategies to use at home. Some parents also described drawing on their experience of managing their own anxiety as helping them to provide support for their child (‘*I said to Peter about sort of when you sort get worried deep breaths and letting it out and counting to ten the things sort of I’d been told when I’d had panic attacks and things in the past*’ [ID.1020]).

It was evident however that some parents lacked confidence in their ability to manage their child’s anxiety or perceived their difficulty managing their child’s anxiety as a weakness:

*‘because we’re not very strong or we’re still not really sure how to deal with it*’ [ID.2009]

*‘because as a parent there’s nothing worse than thinking I’ve done everything I can and I can’t help my child’* [ID.1212]

and this prompted them to recognise the need to seek professional help. Notably parents highlighted that the professional support required included a need for support for parents to enable them to provide appropriate support for their child *(‘actually the parents should have help too to help the children I think in the first place’* [ID.1091]).

2.3 Parent considers and prioritises help seeking

It was apparent that some parents went through a period of contemplation where they considered the possibility of seeking professional help (‘*I don’t know erm I’ve said loads of time maybe I should get him some help or some counselling*’ [ID.1131]). Several parents referred to the busyness of life as making it hard for families to devote time to seriously consider or prioritise help seeking:

*‘you just get get so caught up in the humdrum of just daily life…as a parent it’s really difficult to try and juggle and you know just set that time aside to kind of go right ok do you know what we’re going to sit down and we’re going to deal with it with this’* [ID.38].

Similarly, one parent specifically referred to a reduction in other family demands as providing an opportune time to ‘prioritise’ seeking professional help; and other parents identified the opportunity to take part in this study as a prompt to consider or ‘focus’ on help seeking (‘*it was always just bubbling along and causing quite a bit of stress but never actually being looked at in the face….this project allowed* [that] *to happen*’ [ID 2009]). Equally, among parents who had sought professional help, a sense of desperation seemed to elevate help seeking from a possibility to a priority (‘*until it’s screaming you in the face people don’t react and you wait til the worst possible moment to go shit we need to do something about this*’ [ID.1228]).

2.4 Parent willingness to seek help

Parents’ views surrounding i) anxiety treatment; ii) their child’s willingness to seek help; iii) and sharing concerns with other people each influenced their willingness to seek help from professionals for their child’s anxiety. Parents varied in the extent to which they considered professional support or treatment for anxiety difficulties in children as appropriate or beneficial. Some parents described the potential benefit of professional support, either provided through school or by specialist therapists or psychologists; while others were more sceptical (‘*it’s really hard to make someone think differently about certain situations’* [ID1131]).

Some parents expressed concerns that talking to children about anxiety and mental health may trigger or heighten their anxiety or felt that this may have happened with their child (‘*I think sewed a little seed for Sally in her brain erm … the more negatives you give kids the more they’re gonna tap into it and and fuel the fire*’ [ID.1228]). For some parents, either their own or others’ experience of receiving professional support for mental health difficulties influenced their perceptions of the potential benefits for child anxiety:

‘*I’m not totally confident that any of it really helped, there was just something in me that suddenly went right I’ve had enough of being doing this to myself’* [ID.1020])

‘*they did go and get help and she’s had all sorts of counselling and everything else and thankfully after a year later with all this help she’s she’s you know more or less back to how she was before*’ [ID 2011]

Some parents emphasised the importance of a child’s willingness and acceptance of the need for professional support; while others attached less importance to the child’s view or their involvement in the decision making process (‘*where she’s involved obviously I’ll discuss it with her and kind of help her prepare* [but] *I’m not going to discuss discuss all the things*’ [ID 1036]). Several parents expressed concerns that receiving support from professionals would be anxiety provoking for their child (*‘to take her out which it probably will be during school hours to see this psychologist it it will give her a huge amount of anxiety’* [ID.2009]); and some speculated that their child’s reluctance was linked to concerns surrounding what other people would think.

Parents varied in their overall openness or reluctance to share concerns about their child’s anxiety with both family and friends, and with professionals. Interestingly, parent perceptions surrounding the stigma associated with anxiety and mental health seemed important determinants of their openness (or reluctance) to share concerns both informally and with professionals. Parents referred to common negative perceptions associated with anxiety and mental health and expressed concerns surrounding negative consequences for their child if they were to talk to other people about their child’s difficulties:

*‘it is viewed as a weakness it’s a bit liked depression you know it’s a mental health issue and it doesn’t matter how you dress it up or you know all the campaigns or whatever it will always be viewed as a weakness’* [ID.38]

*‘it’d be straight round the school within 30 seconds and she’ll be she’ll be you know outcast as the weirdo within the group’* [ID.1228]

Parents also expressed concerns surrounding other people blaming them as the parent:

*‘you don’t talk about it sometimes because you think I can’t deal with the negative feedback and you’re going to start saying it’s me’* [ID.2]

*‘it’s not wanting to look like you’re doing the wrong thing parenting wise’* [ID.1131]

They also described feeling a sense of failure or blaming themselves. This type of self-stigma deterred some parents from both talking to friends and family (*‘it’s not the kind of thing you want to talk about with your friends you know being honest with you…*[a] *sense of failure perhaps and that I don’t know what to do with her*’ [ID.7]) and from raising concerns with professionals (‘*if it’d got that far then I’d done something wrong I may not have done but that would be the response I know what my response would be I would be upset concerned what have I done wrong’* [ID.3]).

Parents referred to the importance of raising public awareness and understanding of anxiety and mental health difficulties in children, and some also specifically identified the potential benefit of online resources to allow parents to seek information and advice in private (‘*people would probably want to do it through the internet at home by themselves cos they at that stage because they wouldn’t necessarily want to be broadcasting it’* [ID.7]).

2.5 Professional guidance

As well as highlighting a role for professionals in helping parents identify a child’s difficulties, some parents also identified a role for professionals in helping parents determine whether their child needed support from professionals to help address their anxiety or not. Parents who did not feel their child needed professional support sometimes anticipated that this view would change if a professional recommended getting professional support (‘*if somebody said to me I really do think your child suffers from either mild or whatever moderate anxiety and would benefit from some sort of help then I would be right ok where do I get it from then what do I do*’ [ID.2011]).

3. Parent contacts professionals

3.1 Type of impairment

For some parents, the fact that their child’s anxiety was not evident at school or impacting on their school work deterred them from seeking advice from teachers (‘*she doesn’t do that at school…so I suppose now I wouldn’t tell teachers*’ [ID 7]). In contrast, those parents who were in regular contact with teachers about other difficulties their child was facing (e.g. learning or behavioural) also sought advice from teachers about their child’s anxiety.

3.2 Parent help seeking knowledge

Many parents expressed a degree of uncertainty surrounding what professional support was available and who best to contact for help or advice (‘*you see it’s knowing where to look for the help who do you ask erm’* [ID.2011]). Several parents also emphasised that they felt there was not a clear first point of contact who could signpost families to the available sources of support. Similar to the earlier recognition stages, some parents described seeking advice about where and how to seek professional help from friends, family, other parents, and colleagues, and through using the internet. Parents’ personal and professional experience also informed the extent of their help seeking knowledge:

*‘I don’t think I know a lot about it erm help that’s available erm… you know I haven’t br-schooled or brought up in England with the systems and everything that’s available it could just be that I’m not aware of what’s available out there just because I’m from another country’* [ID.1036]

*‘I worked closely with CAMHS* [Child and Adolescent Mental Health Services] *anyway through my work and I’d spoken to a colleague who worked for CAMHS’* [ID.1205]

Parents also described the importance of identifying appropriate sources of professional support, and some parents lacked confidence in their ability to make judgements about whether particular types of support were appropriate or not. Parents described using the internet as a source of information and the role of online resources, but several referred specifically to facing difficulties judging the credibility of online information:

*‘People say oh you can google it but you don’t you need to know what you are googling don’t you and what’s going to be good you know’* [ID.2]

*‘I’ve started looking on the internet and stuff but it’s there’s so much you know crap really on there that you don’t know where to trust or anything’* [ID.1131]

In a similar vein, a few parents highlighted the need for guidance for parents on the help seeking process, and about the support and resources that are available:

*‘if there was kind of a map of how do you this…have something like that and go I’ve completed this whatever survey I’ve hit sixteen …and my kind of next port of call is to call you and see what help there is either within the GP surgery or externally where could I go where would you recommend I go’* [ID.38]

3.3 Perceived role and expected response from teachers and GPs

Some parents considered a GP to be an appropriate point of contact for parents concerned about their child’s anxiety (‘*I just assume that you have to go the doctor to try to get some help*’ [ID.1020]); whereas others felt that it was not appropriate to seek advice from a GP for emotional difficulties (‘*it’s not really a medical thing is it…it’s not something that you would go to the GP for I mean if your child has tummy ache and you know it’s not not because of a physical thing it’s more emotional thing’* [ID.1036]). Similar contrasting views were expressed in relation to a teacher’s role as a point of contact for parents of children with anxiety difficulties:

‘[the school] *that would be sort of my natural way of looking for some some advice’* [ID.1091]

*‘she’s a teacher she’s there to teach…Yeah I’d see it as though I’m offloading my problems onto somebody that’s not what they’re there to listen to’* [ID.7]

As well as signposting families to external sources of professional support, some parents also referred to the school’s role as a direct source of support to help a child manage their anxiety (‘[the teacher] *giving them strategies to help them get through it*’ [ID 2009]).

It was equally apparent that parent perceptions surrounding the anticipated response from GPs/teachers impacted on their decision to contact (or not) a professional for help or advice. Several parents expressed reservations about raising concerns with a teacher or GP because they felt their concerns would be dismissed (‘*I would worry that I would be fobbed off* [by the GP] *with oh no you know she’s fine and not necessarily taken seriously enough’* [ID 2011]). Others were also hesitant for fear that teachers or GPs may blame them as a parent (‘*you’re thinking do they think you know I should have done this or I should have done that erm’* [ID 1212]). The family’s relationship with the GP and/or teacher, and whether the parent trusted the GP/teacher or not, also seemed to prompt/deter contact with these professionals. For example, the fact that teachers ‘*see your child everyday*’ [ID 1205] and that school is a child’s ‘*familiar environment*’ [ID 1091] were given as reasons to seek help through school. Similarly, while some parents commented that their family did not regularly visit the GP, one parent described that the GP knew her daughter and her history; and was therefore an appropriate point of contact.

3.4 Perceived availability of professional support

Parent perceptions regarding the availability of professional support for children with anxiety difficulties also appeared to play a role in some parental decisions to contact (or not contact) professionals for help or advice. Some parents felt uncertain about whether any appropriate support was available or not, and several referred to anticipated long waiting lists to access support as a deterrent to contacting professionals for help or advice. In contrast, one parent felt confident professional support was available, and did not feel that doubt surrounding the availability of support had deterred them from seeking help.

4. Family receives professional support

4.1 Service criteria

Parents who had sought help described the importance of ‘ticking boxes’ and the need for a child’s difficulties to match strict specialist service requirements (‘*Joseph didn’t quite meet the criteria*’ [ID.1205]), and that less severe difficulties do not meet these criteria. Several parents referred to Child and Adolescent Mental Health Services (CAMHS) prioritising cases where there was a risk of self harm or suicide, and one parent attributed her daughter’s quick access to a CAMHS service to her ‘priority’ status. In relation to receiving support through school, parents described a similar need for difficulties to meet school criteria, with priority given to children with behavioural difficulties (‘*there were always children that were considered erm a bigger concern cos Jasmine’s behaviour wasn’t in disruptive in in school she didn’t affect anyone else*’ [ID 53])

4.2 Parent help seeking skills

The need for parental perseverance, and the importance of not giving up and repeated contact with different professionals, was evident among those families who had received support from services. Equally, parental resilience in the face of dismissive attitudes among professionals also seemed crucial in some cases (‘*yes when everyone kept saying it’s your parenting I kind of thought why am I even bothering why am I following this if I’m not actually going to get anywhere’*[ID.1051]).

One parent also emphasised the importance of a parent’s communication skills and how parents communicate concerns with professionals (*‘there’s no point getting people’s backs up and going in all guns blazing you’ve got to kind of go nicely nicely and kind of get on side with people to try and get help and ask the right questions in the right way to receive the right response*’ [ID. 53]). This parent also felt that as a health professional she was taken more seriously by professionals. Parents who had contacted professionals also described the need for parents to demand or shout loudly for a referral from their GP. The potential benefit of parents preparing for discussions with professionals and gaining a good understanding of the available services was also clear *(‘before I went to see her I thought I need to be clear on what I’m asking her to help with’* [ID.2009]).

4.3 Professional response

Professionals were seen as having a key role in enabling or preventing a family from obtaining support to help with the child’s anxiety. Professional recognition of the child’s need for services was identified as a requirement for receiving professional support and as a means to access additional support (‘*it wasn’t until I got the diagnosis from CAMHS and I could say to them* [school] *look this is what they’ve said she’s got that they would take any notice really*’ [ID.1212]). Parents referred to the importance of the level of knowledge and skills among professionals, both in relation to identifying a child’s difficulties and in relation to an ability to disseminate information about available support and services, with several highlighting the need for training for school staff. The importance of communication skills among professionals was also evident, and parents identified the need for professionals to communicate well with both the child and the parent. Parents referred to professionals dismissing their concerns or feeling blamed by professionals (*‘there’s a lot of erm assumptions that it’s to do with my parenting there seems to be a lot of oh well you know it’s obviously you’re not parenting her in the right way yeah there was a lot of offering of various parenting erm courses’* [ID.2]). One parent emphasised the importance of feeling listen to by professionals (*‘If people actually listened to what I was saying rather than just trying to fob me off’* [ID.1051]), and another identified the key role of talking to the right individual, and someone who responds positively, in order to successfully access professional support.

4.4 Service provision

Parents who had sought help described a lack of available professional support, and high demands on available services as barriers to receiving treatment (‘*the doctor said that there was a you know a waiting time anyway erm for for that sort of help erm’* [ID.1020]). Parents who had not sought help also anticipated that if they did seek help, a lack of available support would present a hurdle to obtaining support (‘*my fear would be that you know your child needs help but then you know it all falls apart… there’s nobody within a hundred mile radius that can help you with it for six months*’ [ID.2011]). Among parents with a child who had been referred to CAMHS, the complex and lengthy referral system was also described as a significant hurdle to treatment access. Parents similarly experienced or anticipated limited provision of support within schools to help children with anxiety difficulties (‘*I guess school can help support them but to be honest I don’t think they’ve got the time or resources’* [ID.1020]). Some parents considered private services as a potential means to avoid waiting lists, but cited the cost of these services as a barrier. One parent however also felt that suitable private services were not ‘*readily available*’ [ID.38].
